# Supplementary material for: The Systems Biology Research Tool: evolvable open-source software
Source: BMC Syst Biol. 2008 Jun 29;2:55. doi: 10.1186/1752-0509-2-55 (PMC2446383; doi:10.1186/1752-0509-2-55)
Supplement: Additional file 1 — SBRT Archive. An archive of the current version of the Systems Biology Research Tool. [file 1752-0509-2-55-S1.zip › sbrt-1.4.0/doc/developers_guide/api/sbrt/kernel/MonitoredProcess.html]

MonitoredProcess


|  |  |  |
| --- | --- | --- |
| **Overview** \| **Package** \| **Class** \| **Use** \| **Tree** \| **Deprecated** \| **Index** \| **Help** | |  |
| **PREV CLASS**   NEXT CLASS | **FRAMES**    **NO FRAMES**     **All Classes** |  |
| SUMMARY: NESTED \| FIELD \| CONSTR \| METHOD | DETAIL: FIELD \| CONSTR \| METHOD |  |


---


## sbrt.kernel Interface MonitoredProcess<R>

**Type Parameters:**: `R` - the type of result an instance of this interface produces.

**All Superinterfaces:**: KernelProcess<R>

**All Known Implementing Classes:**: CatalystDeletionProcess, CdHarFluxVectorProcess, CdHarProcess, CvCatalystDeletionProcess, CvFbaOptimizationExperiment, CvFluxIntervalProcess, CvObjectiveFunctionProcess, CvRxnDeletionProcess, CycleIdProcess, CycleIdProcess, ExtremeCurrentProcess, FluxActivityProcess, FluxCapProcess, FluxIntervalProcess, FluxomeReducerProcess, FluxPlasticityProcess, IntervalComparison, MultipleCorrelationAnalysis, ObjectiveFunctionAnalysis, PathIdProcess, RandomConstraintsGeneratorV1, RandomEnvGeneratorV1, RandomObjectiveGenerator, RxnDeletionProcess, StoichVariationProcess, SvCdHarFluxVectorProcess, SvInitialPointProcess, VectorComparison

---

``` public interface MonitoredProcess<R> extends KernelProcess<R> ```

This interface is used to represent monitored
processes.

**Author:**
:   This interface was written and documented by
    Jeremiah Wright while in the Wagner lab.

---

| **Method Summary** | |
| --- | --- |
| `ProgressMonitor` | `getProgress()`             Returns a progress monitor for this process. |

**Methods inherited from interface sbrt.kernel.KernelProcess**

`run`

**Method Detail**

### getProgress

```
ProgressMonitor getProgress()
```

:   Returns a progress monitor for this process.

    :   **Returns:**: a progress monitor for this process.


---


|  |  |  |
| --- | --- | --- |
| **Overview** \| **Package** \| **Class** \| **Use** \| **Tree** \| **Deprecated** \| **Index** \| **Help** | |  |
| **PREV CLASS**   NEXT CLASS | **FRAMES**    **NO FRAMES**     **All Classes** |  |
| SUMMARY: NESTED \| FIELD \| CONSTR \| METHOD | DETAIL: FIELD \| CONSTR \| METHOD |  |


---
